# Engraftment of Transplanted Buccal Epithelial Cells onto the Urethrotomy Site, Proven Immunohistochemically in Rabbit Model; a Feat to Prevent Urethral Stricture Recurrence

**DOI:** 10.1007/s12015-022-10466-1

**Published:** 2022-10-28

**Authors:** Akio Horiguchi, Masayuki Shinchi, Kenichiro Ojima, Yusuke Hirano, Toshihiro Kushibiki, Yoshine Mayumi, Kosuke Miyai, Ichiro Miura, Masaru Iwasaki, Vaddi Suryaprakash, Rajappa Senthilkumar, Senthilkumar Preethy, Shojiro Katoh, Samuel J. K. Abraham

**Affiliations:** 1 Department of Urology, National Defence Medical College, Tokorozawa, Saitama Japan; 2 Department of Medical Engineering, National Defence Medical College, Tokorozawa, Saitama Japan; 3 Departmet of Basic Pathology, National Defence Medical College, Tokorozawa, Saitama Japan; 4 Department of Clinical Laboratory, Hokkaido Institutional Society, Obihiro Hospital, Obihiro, Hokkaido Japan; 5 Department of Human Pathology, Juntendo University, Tokyo, Japan; 6 Center for Advancing Clinical Research (CACR), University of Yamanashi -Faculty of Medicine, 1110, Shimokato, Chuo, Yamanashi 409-3898 Japan; 7 Department of Urology, Yashoda Hospitals, Raj Bhavan Rd, Matha Nagar, Somajiguda, Hyderabad, Telangana 500082 India; 8 The Fujio-Eiji Academic Terrain (FEAT), Nichi-In Centre for Regenerative Medicine (NCRM), PB 1262, Chennai, 600034 Tamil Nadu India; 9 Edogawa Evolutionary Lab of Science (EELS), 2-24-18, Higashi-Koiwa, Edogawa, Tokyo, 133-0052 Japan; 10 The Mary-Yoshio Translational Hexagon (MYTH), Nichi-In Centre for Regenerative Medicine (NCRM), PB 1262, Chennai, 600034 Tamil Nadu India; 11 Division of Research & Development, JBM Inc, Tokyo, Japan; 12 Antony- Xavier Interdisciplinary Scholastics (AXIS), GN Corporation Co. Ltd, 3-8, Wakamatsu, Kofu, Yamanashi, 400-0866 Japan; 13 University of Yamanashi - School of Medicine, Chuo, Japan

Dear Editor,

Urethral strictures characterized by narrowing of the urethral lumen is a common disorder in men caused by inflammation, infection or trauma. Less invasive transurethral procedures in managing short segment strictures such as urethral dilation and direct vision internal urethrotomy (DVIU) while offer a transient relief, buccal mucosal graft urethroplasty for long anterior urethral stricture, that are not amenable to anastomotic urethroplasty reported with high long-term success rate is highly invasive. BEES-HAUS (buccal epithelium expanded and encapsulated in scaffold, hybrid approach to urethral stricture) is a minimally invasive approach of transplanting buccal mucosal cells into the urethra after urethrotomy was found efficacious in a clinical study [1] followed by reproduction of cell engraftment in rabbits [2] proven morphologically. Herein, we report the proof of engraftment of transplanted buccal mucosal cells over the urothelium at the urethrotomy site by immunohistochemistry markers positive for buccal epithelium, while negative for urothelium.

Six Japanese white male rabbits (weight: 2.5–3.5 kg) were used (Ethical approval project number 19011 and 19,066, Institutional Animal Care and Use Committee, National Defence Medical College, Saitama, Japan). After intramuscular anaesthesia with ketamine hydrochloride (35 mg/kg) and xylazine (5 mg/kg), stricture was created by electrocoagulation. Stricture formation was confirmed by retrograde urethrography and urethroscopy after 14 days, when buccal tissue of the rabbits was harvested and subjected to cell processing as reported [1, 3]. Briefly, after enzymatic digestion (Dispase II), the epithelial layer was peeled, minced, digested (1 ml Accutase), which after filtration (EASYstrainer), centrifugation (400 g ∗ 5 min ∗ 20° C) and counting were cultured in two groups, i. with thermo-reversible gelation polymer (TGP) scaffold, (3D-TGP) and ii. without TGP, (2D) for 7 days and then cells of these two groups were mixed as cells-TGP cocktail for transplantation. Prior to transplantation, through urethrotomy, the fibrous strands at stricture site were released, a catheter was placed [2] and the cells-TGP cocktail, was injected between the catheter and the urethral lumen. Catheter was removed on day 3 and on day 7 after transplantation, a urethrogram was taken, animals were sacrificed, and the urethra were evaluated histologically.

Haematoxylin and eosin staining was performed. Immunohistochemical staining was performed using CK14, a positive marker for buccal mucosal epithelial cells and GATA-3, a positive marker for urothelium. For CK14, the first staining (enzyme antibody; Labeled Strept Avidin–Biotin) with normal sensitivity and the second staining (Enzyme Antibody Multimer AP; ultraView RED) of higher sensitivity were performed (primary antibody: Abcam Mouse Monoclonal Anti-CK14(LL002) Code: ab77684. Secondary antibodies: 1^st^ stain- DAKO Goat Anti-Mouse IgG / Biotinylated code: E0433; 2^nd^ stain- Abcam Goat Anti-Mouse IgG / Alkaline Phosphatase code: ab7069). For GATA-3, Immuno-peroxidase staining was done (Ventana Benchmark XT automatic slide staining system). Formalin-fixed paraffin-embedded tissue was cut to a thickness of 4 µm. The sections were deparaffinized, pre-treated (CC1, Ventana Medical Systems, USA), reacted with the primary antibody, and visualized with the iView DAB detection kit (Ventana Medical Systems, USA). Counterstaining was performed using haematoxylin and Bluing Reagent (Ventana Medical Systems, USA). The GATA3 antibody (Mouse Monoclonal anti-GATA3, clone: L50-823,) was diluted 1/100 and used with the iVIEW DAB detection kit; (both Ventana Medical Systems, USA) and the endogenous biotin blocking kit (Ventana Medical Systems, USA) using biotin-labelled goat anti-mouse as the secondary antibody (E0433 Dako, Agilent Pathology Solutions, USA).

All the animals developed urethral stricture at the electrocoagulation site confirmed by urethroscopy and retrograde urethrogram [2]. Figure 1A and B shows the post-electrocoagulation stricture specimens of urethra of rabbits. In our earlier study, urethral stricture site was examined by longitudinal sectioning of the urethra, that showed fibrous strands across, which on dissection revealed underlying hardened erythematous area, resembling the pathology of human urethral stricture [2]. In this study, the whole urethra, were sent for immuno-histochemical staining, with a suture placed externally, corresponding to the internal stricture site (Fig. 1C), from which sections were stained for H&E, CK14 and GATA3 (Fig. 1D-L). HE staining showed the superficial layer of the transplanted portion having features of oral mucosa with papillae and stratified squamous epithelium (Fig. 1D, G, J). The CK14 was positive on the lumen covered with the oral squamous epithelium (Fig. 1E, H, K). The GATA3 was negative in the region, that was positive for CK14, while remaining urothelium stained positively with GATA3 (Fig. 1F, I, L) doubly confirming the engraftment.

**Fig. 1:**
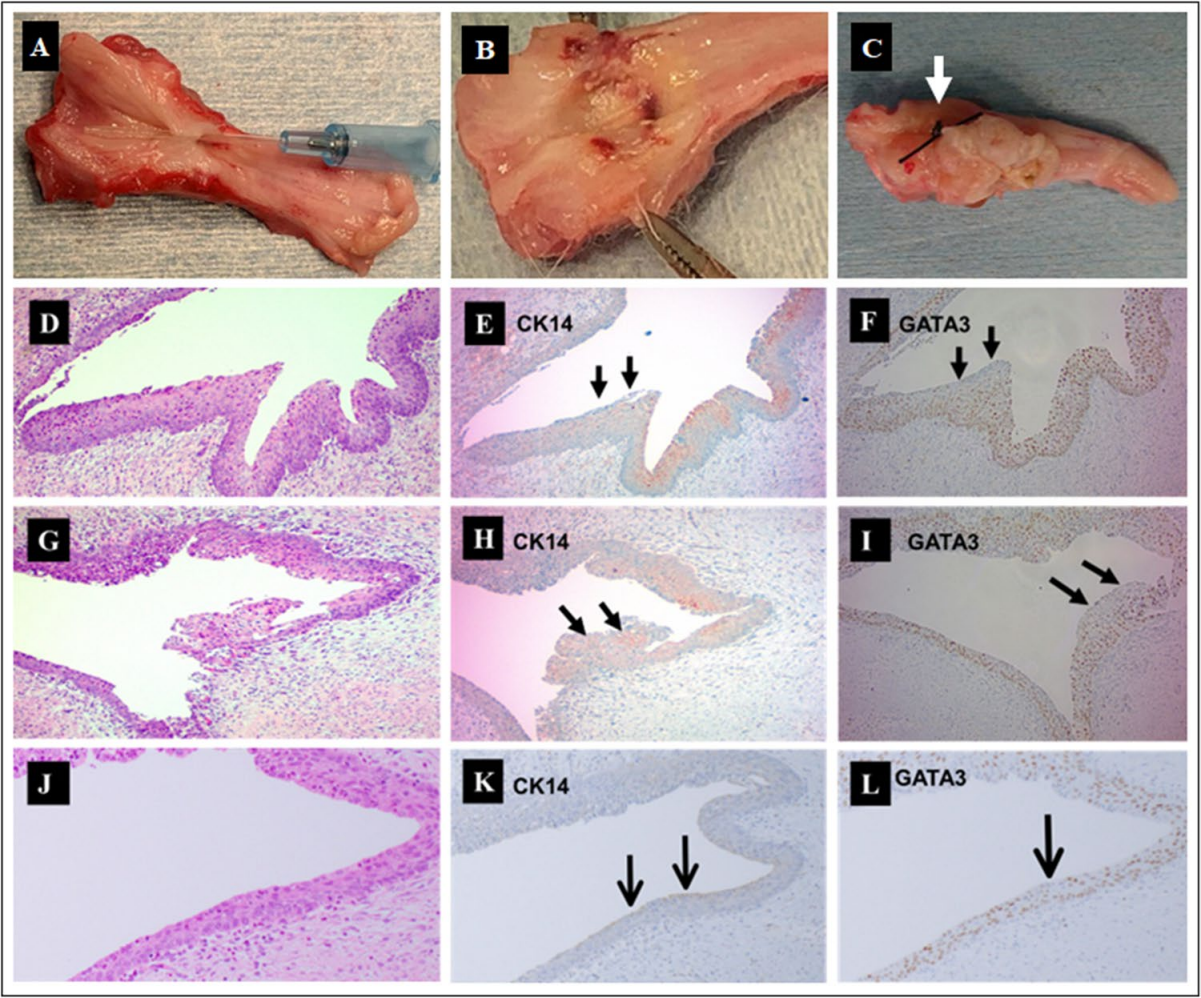
**A & B** images show the stricture-induced urethra of rabbits. Visual examination of the harvested specimens after longitudinally split opening the urethra revealing fibrous strands in the coagulation-induced stricture site (**A**); A cannula across a fibrous strand in **A**; In another specimen, underneath the fibrous strands, marked inflammation and hardened stricture site are seen in **B**. **C**. Post-transplantation, the harvested urethra as a whole, were sent for immuno-histochemical staining, with a suture placed externally (White arrow), indicating the corresponding site of stricture site internally, from which sections were evaluated for positive and negative staining with GATA3 and CK14. **D ~ L:** Cross sections of the urethra at the site of urethrotomy; **D**, **G** and **J** show HE images, **E**, **H** & **K** of CK 14 stain, positive marker for epithelium and **F**, **I** & **L** of GATA3 stain, a positive marker for urothelium. Black arrows indicate transplanted CK14 positive and GATA3 negative cells, which are indicative of transplanted squamous epithelium

Male urethral stricture, due to trauma (20%), iatrogenic (45%) and idiopathic aetiology (30%) [1, 2], affects men of all ages, leading to voiding difficulty and infertility. Short segment stenosis is tackled by internal urethrotomy and dilatation, whose stricture recurrence rate is 37% in 4.5 months. Such short segment stricture at disease onset, over time, progresses to longer segment stricture, for which buccal mucosal graft urethroplasty is the standard treatment, an invasive, technically demanding procedure with a long learning curve. Cell therapy-based approaches such as liquid buccal mucosa graft endoscopic urethroplasty [4], and autologous adult live cultured buccal epithelial cells (AALBEC) [5] despite reporting a positive outcome, use of biological materials in those procedures carry potential risks of contamination. The BEES-HAUS is less invasive [1], uses a synthetic scaffold, with efficacy in a pilot clinical study with more than three-years recurrence-free interval [1] and proven in animal study [2] for its easy reproducibility and cell engraftment. Now, the engraftment by delineation of buccal mucosal cells over the damaged urothelium having been proven immunohistochemically covering the damaged urethral site of injury, which would prevent an inflammatory reaction induced proliferation of the underlying sub-epithelial cells leading to fibrosis, this we consider has accomplished the most important step against stricture recurrence. Any interventional treatment for urethral stricture either dilatation or urethrotomy or both leaves an open injury without an epithelial coverage exposing the sub-epithelial tissue to urine, that provokes inflammation leading to recurring of stenosis. Proven cell engraftment covering the open wound of urethrotomy, amidst pathology of a stricture with fibrous adhesions in this study, makes us recommend the BEES HAUS procedure as the first choice of treatment with potential to yield long-term stricture-free endurance of the male urinary tract.

## Data Availability

The data supporting the findings of this study are available within the article.

## References

[1] Vaddi SP, Reddy VB, Abraham SJ (2019). Buccal epithelium Expanded and Encapsulated in Scaffold-Hybrid Approach to Urethral Stricture (BEES-HAUS) procedure: A novel cell therapy-based pilot study. International Journal of Urology.

[2] Horiguchi A, Ojima K, Shinchi M, Kushibiki T, Mayumi Y, Miyai K, Katoh S, Takeda M, Iwasaki M, Prakash VS, Balamurugan M, Rajmohan M, Preethy S, Abraham SJ (2021). Successful engraftment of epithelial cells derived from autologous rabbit buccal mucosal tissue, encapsulated in a polymer scaffold in a rabbit model of a urethral stricture, transplanted using the transurethral approach. Regenerative Therapy.

[3] Katoh S, Rao KS, Suryaprakash V, Horiguchi A, Kushibiki T, Ojima K (2021). A 3D polymer scaffold platform for enhanced in vitro culture of Human & Rabbit buccal epithelial cells for cell therapies. Tokai Journal of Experimental and Clinical Medicine.

[4] Scott KA, Li G, Manwaring J, Nikolavsky DA, Fudym Y, Caza T, Badar Z, Taylor N, Bratslavsky G, Kotula L, Nikolavsky D (2020). Liquid buccal mucosa graft endoscopic urethroplasty: A validation animal study. World Journal of Urology.

[5] Kulkarni SB, Pathak H, Khanna S, Choubey S (2021). A prospective, multi-center, open-label, single-arm phase 2b study of autologous adult live cultured buccal epithelial cells (AALBEC) in the treatment of bulbar urethral stricture. World Journal of Urology.

